# Attention-based multi-label neural networks for integrated prediction and interpretation of twelve widely occurring RNA modifications

**DOI:** 10.1038/s41467-021-24313-3

**Published:** 2021-06-29

**Authors:** Zitao Song, Daiyun Huang, Bowen Song, Kunqi Chen, Yiyou Song, Gang Liu, Jionglong Su, João Pedro de Magalhães, Daniel J. Rigden, Jia Meng

**Affiliations:** 1 Department of Mathematical Sciences, Xi’an Jiaotong-Liverpool University, Suzhou, PR China; 2 Department of Biological Sciences, Xi’an Jiaotong-Liverpool University, Suzhou, PR China; 3 Department of Computer Sciences, University of Liverpool, Liverpool, United Kingdom; 4 Institute of Systems, Molecular and Integrative Biology, University of Liverpool, Liverpool, United Kingdom; 5 Laboratory of Ministry of Education for Gastrointestinal Cancer, School of Basic Medical Sciences, Fujian Medical University, Fuzhou, PR China; 6 School of AI and Advanced Computing, XJTLU Entrepreneur College (Taicang), Xi’an Jiaotong-Liverpool University, Suzhou, PR China; 7 Institute of Ageing and Chronic Disease, University of Liverpool, Liverpool, United Kingdom; 8 AI University Research Centre, Xi’an Jiaotong-Liverpool University, Suzhou, PR China

**Keywords:** RNA, Computational models, Machine learning

## Abstract

Recent studies suggest that epi-transcriptome regulation via post-transcriptional RNA modifications is vital for all RNA types. Precise identification of RNA modification sites is essential for understanding the functions and regulatory mechanisms of RNAs. Here, we present MultiRM, a method for the integrated prediction and interpretation of post-transcriptional RNA modifications from RNA sequences. Built upon an attention-based multi-label deep learning framework, MultiRM not only simultaneously predicts the putative sites of twelve widely occurring transcriptome modifications (m^6^A, m^1^A, m^5^C, m^5^U, m^6^Am, m^7^G, Ψ, I, Am, Cm, Gm, and Um), but also returns the key sequence contents that contribute most to the positive predictions. Importantly, our model revealed a strong association among different types of RNA modifications from the perspective of their associated sequence contexts. Our work provides a solution for detecting multiple RNA modifications, enabling an integrated analysis of these RNA modifications, and gaining a better understanding of sequence-based RNA modification mechanisms.

## Abstract (web summary)

RNA modifications appear to play a role in determining RNA structure and function. Here, the authors develop a deep learning model that predicts the location of 12 RNA modifications using primary sequence, and show that several modifications are associated, which suggests dependencies between them.

## Introduction

Post-transcriptional RNA modifications increase the structural and functional diversity of RNA molecules and regulate all stages of RNA life^1^. Precise identification of RNA modification sites is therefore of crucial importance to understanding the functions and regulatory mechanisms of various RNAs. More than 100 different types of RNA modifications have been identified^2^, and among them, *N*^6^-methyladenosine (m^6^A) is the most common eukaryotic mRNA modification. M^6^A occurs on nascent pre-mRNA, regulating its stability and translation. It is involved in many biological processes such as the circadian clock, differentiation from naïve pluripotency, and the heat shock response. It also plays various roles in disease pathogenesis such as carcinoma, breast tumor, gastric cancer, and anti-tumor immunity. Besides m^6^A, there are also a number of RNA modifications with crucial biological functions. For instance, *N*^1^-methyladenosine (m^1^A) can block the Watson-Crick interface and is vital for tRNA stability and the replication of HIV-1.

To date, a number of computational approaches have been proposed for in silico prediction of RNA modification sites from the primary RNA sequences, including: the iRNA toolkits^3–11^, SRAMP^12^, DeepPromise^13^, WHISTLE^14^, Gene2vec^15^, m6A-Atlas^16^, RMDisease^17^, PEA^18^, PPUS^19^, BERMP^20^, m5Upred^21^, and m6AmPred^22^. Special attention has also been paid to the prediction of RNA modifications in introns^23^, lncRNAs^24^ as well as various tissues and cell lines^25–27^. Together, these works greatly advanced our understanding of the localization of multiple RNA modification types in different species under various conditions^28^. However, existing approaches suffered from the following limitations.

Firstly, most existing studies only focused on a single RNA modification type, mainly m^6^A, but failed to support multiple RNA modifications simultaneously through an integrated predictive model. Therefore, the study of the interplay between different modifications is limited. The iRNA toolkit^3–11^ developed primarily by Chen, Lin and Chou are the earliest as well as the most comprehensive approaches that support the prediction of various RNA modifications from RNA sequences and have been widely adopted as the gold standard for benchmarking the performance of different RNA modification prediction methods. However, the iRNA toolkit was presented in the form of multiple independent studies, each targeting a single modification. The iMRM web server^29^ was aimed to support five RNA modifications simultaneously with a friendly web graphical user interface; however, it is still based on five independent binary predictors corresponding to the five RNA modifications, respectively, without considering potential interactions among different modifications. Given the intrinsic biochemical and biophysical properties of different RNA modifications, the predictive framework established for one type of modification can often be conveniently migrated for the prediction of another modification. It is thus beneficial and efficient to test the computational framework on multiple RNA modifications simultaneously. Very recently, by taking advantage of the generative adversarial network (GAN), the MR-GAN approach was developed to predict eight RNA modifications^30^. However, some of the modifications supported may be rare modifications, such as m^1^G (only 29 sites), m^2^G (only 59 sites), and D (only 162 sites)^30^, whose wide occurrence in human transcriptome has not yet been confirmed. Given a large number of negative (non-modifiable) sites of such rare RNA modifications, the sequence-based prediction is likely to produce a substantial proportion of false-positive predictions in practice and should be used with extra caution.

Secondly, most existing works relied on a limited amount of data from a single source (a single database or dataset generated from a single experiment), failing to fully take advantage of the available epi-transcriptome information. For example, the wide occurrence of m^5^U modification has been previously confirmed with thousands of m^5^U sites reported by two different approaches (miCLIP and FICC-seq)^31^. Nevertheless, MR-GAN used only 30 sites for its training, which is likely to seriously limit its predictive capability for this specific modification. In addition, substantial discrepancies have been reported previously between different epi-transcriptome profiling technologies, e.g., for m^5^C^32^ and Ψ^33^. Thus, it is crucial to take advantage of the data generated from multiple orthogonal technologies to minimize the potential technological bias whenever such datasets are available.

Thirdly, most of the work in the field, such as SRAMP^12^ and iMRM^29^ focused on prediction accuracy but failed to provide a clear and intuitive interpretation of their prediction results. Although some existing approaches carefully interpreted their trained predictive model^27,34^, to the best of our knowledge, none of the existing works provided insights into their decision-making process for individual predictions. Recent advances in interpretable RNA/DNA models enabled the extraction of low-level CNN kernels and visualizing them as position weight matrices (PWM). These patterns, however, provide only vague insights, especially for multiple-layer DNNs, and cannot provide nucleotide level interpretation. However, it remains of significant interest to identify the critical sequence contents that directly contribute to positive RNA modification predictions, which should help facilitate our understanding of the sequence-dependent forming mechanisms of individual RNA modification sites.

Lastly, a predictive framework has not been developed for some RNA modification types such as m^6^Am, even though its base-resolution epi-transcriptome profiling technology miCLIP has been developed, and the profiling data is publicly available.

For these reasons, there is strong motivation to take advantage of state-of-the-art deep learning techniques to develop a unified predictive framework that supports multiple RNA modifications by integrating datasets generated from multiple technologies.

We present here MultiRM, an attention-based multi-label neural network approach for integrated prediction and interpretation of RNA modifications from the primary RNA sequence (or the corresponding DNA sequence). Twelve RNA modification types are supported by our model, including m^6^A, m^1^A, m^5^C, m^5^U, m^6^Am, m^7^G, Ψ, I, Am, Cm, Gm, and Um. To the best of our knowledge, these are the only widely occurring RNA modifications that can be profiled transcriptome-wide with existing base-resolution technologies, which are highly desired characteristics of RNA modification for reliable large-scale prediction. The multi-label architecture of our approach enables accommodation of the shared structure of different modifications while fully exploiting their distinct features. As some modifications are still more abundant than the others, to handle the imbalanced training data issue in multi-label learning, online hard examples mining (OHEM)^35^ and Uncertain Weighting^36^ were utilized. Some widely adopted state-of-art machine learning algorithms XGBoost^37^ and CatBoost^38^ were also implemented as the benchmarks. Importantly, we used the integrated gradient (IG)^39^ and the attention weights^40^ to gain insights into the trained overall model and to explain every individual prediction. Finally, a web server was developed and made freely accessible to serve the research community.

## Results

### The MultiRM framework

**Fig. 1:**
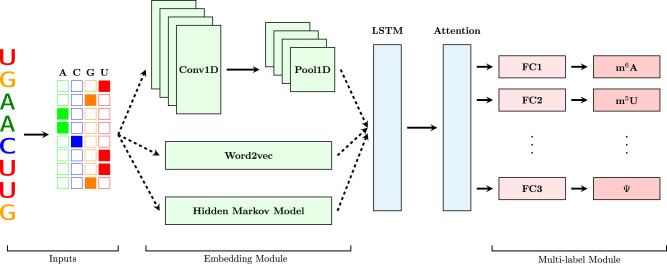
A graphic illustration of the MultiRM model architecture. The model consists of an embedding module and an LSTM-Attention block to extract and learn useful features. Then, features filtered by attention are fed into a multi-label module to predict RNA modifications simultaneously. Conv1D stands for 1D convolutional layer; Pool1D stands for 1D max-pooling layer; FC stands for fully connected layer.

Our framework predicts twelve types of widely occurring RNA modifications using a deep neural network, as shown in Fig. 1. Given a set of base-resolution modifiable sites, MultiRM learns the mapping between the site sequence context and the modification type. Once this mapping is learned, the attention mechanism and IG method enable us to interpret the model and extract the sequence content that contributes the most to the positive prediction, the sequence motif. The proposed integrated model using a multi-label framework is also presumed to benefit learning the underlying association among different RNA modifications.

MultiRM consists of an embedding module representing the input RNA sequences using the inherent short- and long-range interactions among nucleotides. The embedded representation is fed to an LSTM layer to extract the underlying sequence features shared by all modifications. Then, the attention mechanism enables the model to focus on the relevant region of the input RNA sequence for each specific modification type as needed. Finally, the multi-label module containing two fully connected (FC) layers predicts the multiple modifiable sites simultaneously. The framework is trained using a cross-entropy loss enhanced by OHEM and Uncertain Weighting.

### MultiRM performance

The primary purpose of our study is to establish an interpretable predictor that could achieve state-of-the-art accuracy in the identification of multiple widely occurring RNA modifications from the primary RNA sequences.

We firstly tried to optimize the length of the input sequences according to AUC_b_ (b stands for binary). AUC_b_ is the area under ROC curves calculated based on the positive and the corresponding negative samples (e.g., A for m^1^A) of each modification. Using the Word2vec embedding, we evaluated our multi-label model with 21-bp, 51-bp, and 101-bp RNA sequences as the input. As shown in Table 1, the input of the 51-bp sequence obtained the best average performance of all the modifications, and this setting also returned the best performance on six out of the twelve RNA modifications tested. It may be worth mentioning that the 51-bp of the input sequence is also optimal for the XGboost method (Supplementary Table 1).

**Table 1:** AUC_b_ scores of models (w2v + LSTM + attention) with different input length.

Length (bp)	Am	Cm	Gm	Tm	m^1^A	m^5^C	m^5^U	m^6^A	m^6^Am	m^7^G	Ψ	I	Mean	Median
101	**0.7900**	0.8224	0.9108	0.8596	**0.8300**	0.9108	0.9196	**0.8496**	0.8944	**0.6204**	0.8228	0.6040	0.8195	0.8398
51	0.7272	**0.8452**	0.9324	**0.8832**	0.8068	0.9236	**0.9560**	0.8336	**0.9272**	0.6012	**0.8420**	**0.6304**	**0.8257**	**0.8436**
21	0.7276	0.7984	**0.9468**	0.8396	0.7624	**0.9360**	0.9404	0.7848	0.8460	0.6004	0.7800	0.6084	0.7976	0.7916

Please note that our predictive model requires information of neighboring sequences even if they do not form part of the transcript. Bold indicates the best performance in comparison.

Subsequently, to address the unbalanced training data problem, we implemented OHEM, uncertainty weighting (UW), and focal loss on our optimized multi-label model and tested their performance with 51-bp of input sequences. Improved AUC_b_ values based on the optimized model were then achieved, as shown in Table 2. Both OHEM and UW were beneficial to some modifications. For example, OHEM improved the m^7^G prediction by 0.08 of the AUC_b_ score, and UW raised the AUC_b_ for m^1^A by 0.06. After we combined both of them, it helped to enhance the average AUC_b_ score by 0.0145.

**Table 2:** Improving AUC_b_ with techniques that handle imbalanced data problems.

Method	Am	Cm	Gm	Tm	m^1^A	m^5^C	m^5^U	m^6^A	m^6^Am	m^7^G	Ψ	I	Mean	Median
OHEM	−0.0864	−0.0156	**0.0188**	−**0.0028**	−0.0404	**0.0124**	−0.0102	−0.0832	−**0.0344**	**0.0804**	−0.0156	−0.0252	−0.0169	−0.0156
UW	−0.0144	−0.0025	−0.0384	−0.0176	0.0596	−0.0128	−0.0372	0.0128	−0.0924	−0.0032	−0.0204	0.0168	−0.0125	0.0010
OHEM + UW	**0.0620**	**0.0152**	−0.0068	−0.0052	−0.0276	−0.0180	−**0.0080**	**0.0222**	−0.0360	0.0760	**0.0108**	**0.0394**	**0.0103**	**0.0145**
Focal Loss	−0.0064	−0.1208	−0.0612	−0.1192	**0.0132**	−0.0792	−0.0336	−0.0092	−0.0400	−0.0620	−0.1142	−0.0444	−0.0564	−0.0516

Bold indicates the best performance in comparison.

With the optimized settings (51-bp input, UW + OHEM), we then compared the newly developed approach MultiRM with the baseline approaches and other embedding techniques. The optimized hyper-parameters for each model to be compared can be found in Supplementary Table 2. As seen in Table 3, the newly proposed approach MultiRM obtained the best mean and median performance with AUC_b_ of 0.8361 and 0.8581, respectively, and achieved the best performance on six of the twelve RNA modifications considered (Am, Cm, m^5^U, m^6^A, Ψ and I) with an average ranking of 1.667 among the five approaches tested. The widely adopted XGBoost algorithm obtained the best performance on four modifications (Gm, m^1^A, m^5^C, and m^7^G) and achieved the mean and median performance with AUC_b_ of 0.8035 and 0.8122 with an average ranking of 2.25 among the five approaches considered.

**Table 3:** Comparing MultiRM to baseline approaches under AUC_b_.

Model	Am	Cm	Gm	Tm	m^1^A	m^5^C	m^5^U	m^6^A	m^6^Am	m^7^G	Ψ	I	Average AUC_b_	Average Rank
XGBoost	0.6536	0.8124	**0.9500**	0.7608	**0.8604**	**0.9096**	0.9300	0.8120	0.8668	**0.6796**	0.7956	0.6112	0.8035	2.250
CatBoost	0.5880	0.7736	0.6672	0.6436	0.7100	0.7032	0.8684	0.8116	0.7604	0.5528	0.8056	0.5520	0.7030	4.250
CNN + LSTM + Attention	0.6088	0.7872	0.9304	0.8004	0.7548	0.7732	0.8592	0.7272	0.8324	0.4968	0.6452	0.5116	0.7273	4.167
HMM + LSTM + Attention	0.6984	0.8352	0.8942	**0.8916**	0.8404	0.8892	0.9404	0.7472	**0.9276**	0.5284	0.7216	0.5908	0.7921	2.667
MultiRM	**0.7892**	**0.8604**	0.9256	0.878	0.7792	0.9056	**0.9480**	**0.8558**	0.8912	0.6772	**0.8528**	**0.6698**	**0.8361**	**1.667**

Bold indicates the best performance in comparison.

Subsequently, we selected the optimal thresholds for each modification with the largest G-Mean^41^ value based on their respective ROC curves. The corresponding performance evaluation metrics, including Sensitivity (Sn), specificity (Sp), accuracy (Acc), and Matthews correlation coefficient (MCC) for each modification, was calculated and provided in Table 4. The precisions and recalls (PRs) curves and the receiver operating characteristic curves (ROCs) curves of the MultiRM method are provided in Supplementary Figs. 1 and 2. Please refer to Supplementary Table 3 for the performance metrics of MultiRM under the scheme of multi-label classification^42^.

**Table 4:** Performance summary of MultiRM.

Modification	Sn	Sp	Acc	MCC	AUC_b_	AUC_m_
Am	0.72	0.84	0.78	0.56	0.79	0.90
Cm	0.92	0.72	0.82	0.65	0.86	0.97
Gm	0.90	0.88	0.89	0.78	0.93	0.98
Um	0.86	0.78	0.82	0.64	0.88	0.94
m^1^A	0.64	0.80	0.72	0.45	0.78	0.90
m^5^C	0.92	0.78	0.85	0.71	0.91	0.97
m5U	0.98	0.86	0.92	0.85	0.95	0.95
m^6^A	0.82	0.78	0.80	0.60	0.86	0.99
m^6^Am	0.88	0.78	0.83	0.66	0.89	0.97
m^7^G	0.76	0.54	0.65	0.31	0.68	0.97
Ψ	0.92	0.76	0.84	0.69	0.85	0.94
I	0.68	0.72	0.70	0.40	0.67	0.89
Mean	0.83	0.77	0.80	0.61	0.84	0.95
Median	0.87	0.78	0.82	0.645	0.86	0.96

Sensitivity (Sn), specificity (Sp), accuracy (Acc), and Matthews correlation coefficient (MCC) were all calculated under the binary scenario, i.e., with the original nucleotide of the modification as the negative samples. AUC_b_ was computed from the positive and the corresponding negative samples of each modification (with b representing binary), while AUC_m_ was calculated using all other labels, including all other modifications and all the unmodifiable nucleotides as the negative (with m representing multiple).

### Interpretation

So far, the results have emphasized the performance of our method in terms of classification. To gain insights into the driving features behind the predictions, we applied techniques that are capable of providing model interpretability so as to identify key input sequence contents that are significant for predicting RNA modifications (see “Methods”). The sequence contents within the attention have a greater impact on RNA modifications, and mutations within these regions are more likely to lead to the gain or loss of RNA modification sites, as shown in Supplementary Fig. 3.

**Fig. 2:**
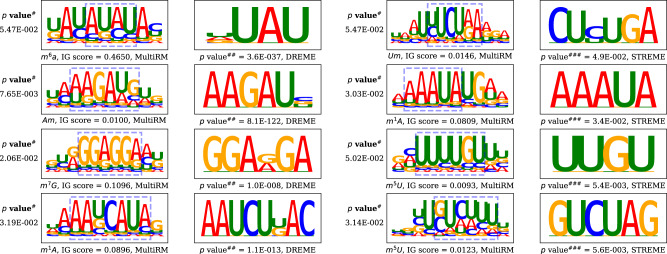
Motif matching. Some motifs identified from MultiRM are similar to those identified from conventional motif analysis (DREME and STREME). $p$ value^#^ was calculated using TOMTOM by utilizing a null model containing MultiRM’s motif columns from all the columns in the set of DREME and STREME motifs. $p$ value^##^ in DREME was calculated by a one-sided fisher’s exact test. $p$ value^###^ in STREME was calculated by a one-sided binomial test. The motifs within the blue dashed anchor boxes were extracted to do pair comparisons. IG scores were calculated by the average of the contribution scores of each nucleotide obtained by the integrated gradients method. Accession codes for the data used to generate this figure are found in Table 5.

Moreover, we aggregated and examined the consensus motifs that played a key role in the MultiRM model. Interestingly, many of them matched the sequence patterns unveiled from conventional motif finding methods DREME^43^ and STREME^44^. To further quantify the similarity between the motifs obtained through MultiRM and DREME/STREME, motif comparison tool TOMTOM^45^ was applied to produce a *p*-value. Sufficient small *p*-values indicate a certain degree of consistency (see Fig. 2). It may be worth mentioning that MultiRM constructed the motifs of an RNA modification using the 6-mers of highest attention weights, which does not necessarily contain the RNA modification site itself. This is consistent with most de novo motif finding algorithms such as DREME^43^ and STREME^44^.

**Fig. 3:**
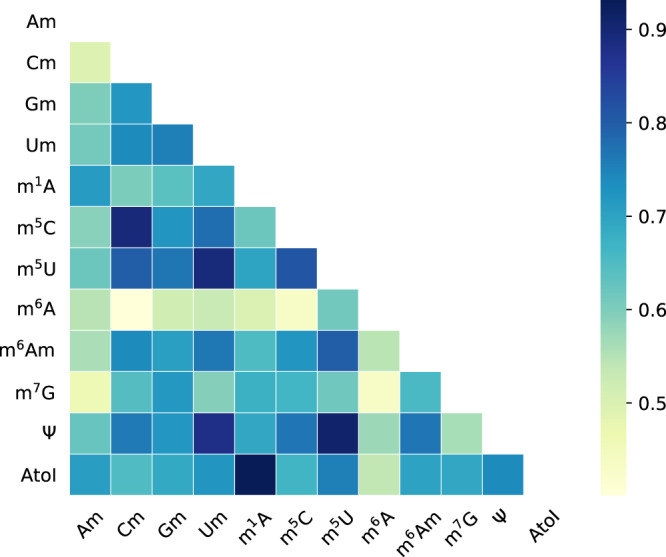
Association of RNA modifications revealed by MultiRM. The RNA modifications considered in MultiRM all show positive relationships among one another, suggesting that there are some regions intensively modified by multiple RNA modifications, which are likely to be key regulatory components for the epi-transcriptome layer of gene regulation. Modifications on the same nucleotide are likely to be more strongly associated with each other, such as m^5^C and Cm (*ρ* = 0.895, *p*-value = 3.81E−36), I and m^1^A (*ρ* = 0.931, *p*-value = 9.57E−45), Ψ and m^5^U (*ρ* = 0.908, *p*-value = 5.47E−39). The two-sided Pearson correlation test was performed using the exact distribution of the sample correlation coefficient. Statistical significance was calculated by the probability that abs(*ρ*′) of a random sample *x*′ and *y*′ drawn from the population with zero correlation would be greater than or equal to abs(*ρ*). The weights used to generate this figure can be found in GitHub’s repository.

A major advantage of the proposed integrated model is the capability to learn the underlying association among different RNA modifications. It was shown previously that there exist clear evolutionary and functional cross-talk among different post-translational modifications of protein^46^ and among different histone and chromatin modifications^47^. Conceivably, such association may also exist at the epi-transcriptome layer among different RNA modifications. To better understand the inherent shared structures among different RNA modifications, we extracted the weights of the feedforward neural network within the attention mechanism. These weights were twelve vectors corresponding to twelve RNA modifications, respectively, and were jointly learned together with all other components of the proposed model. The Pearson’s correlation (*ρ*) of each pair of vectors was calculated to reveal the relevance of two arbitrary RNA modifications unveiled by the integrated prediction model. A surprising finding is the RNA modifications all show strong and significant positive associations among each other, including those, originated from different nucleotides (see Fig. 3). It suggests that there exist regions that are intensively modified by multiple RNA modifications, which are likely to be the key regulatory components for the epi-transcriptome layer of gene regulation. Importantly, the sequence signatures of these key regulatory regions are largely shared among different RNA modifications (including those that modify different nucleotides) and were successfully captured by our model. The most strongly associated modifications originated from the same nucleotide, such as m^5^C and Cm (*ρ* = 0.895, *p*-value = 3.81E−36), I and m^1^A (*ρ* = 0.931, *p*-value = 9.57E−45), Ψ and m^5^U (*ρ* = 0.908, *p*-value = 5.47E−39). Notably, m^6^A showed only mild association with other modifications, implying its relatively special role in post-transcriptional regulation as the most abundant mRNA modification. It is also worth noting that the above analysis did not consider the context-specificity of RNA modifications (or the dynamics of RNA modification across different biological conditions). It does not directly suggest that different RNA modifications should co-occur in the same biological context, or they work with synergetic effects, even though the latter is highly probable as what we have seen in the epi-genetic regulation of histone modifications.

To further validate the above finding, we calculated the pairwise distance between two arbitrary RNA modifications and compared it to the random. Although we could not completely rule out the possibility of experimental bias, e.g., polyA selection, we did observe strong aggregation effects among most RNA modifications considered in this analysis. It is clear that the distance between two arbitrary RNA modifications is likely to be closer than random (Supplementary Fig. 4).

### MultiRM web server

**Fig. 4:**
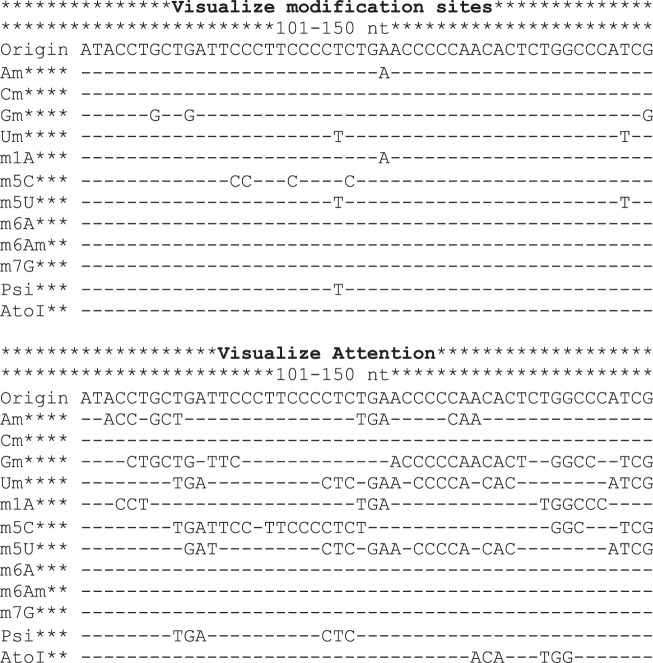
Output of the MultiRM web server. The web server supports site prediction and result interpretation for twelve widely occurring RNA modifications (m^6^A, m^1^A, m^5^C, m^5^U, m^6^Am, m^7^G, Ψ, I, Am, Cm, Gm, and Um) from RNA (or DNA) sequences. The figure shows the predicted RNA modification sites (upper panel) and the sequence components that contributed most to the positive predictions (lower panel) between the 101st and 150th nucleotides of an input sequence. The predicted probabilities, statistical significance, and attention scores of RNA modifications can all be downloaded as separate files from the web server. At the 123^rd^ nucleotide, multiple RNA modifications originated from U were predicted with a *p*-value less than 0.05, which, to some degree, reflected the associations among different RNA modifications unveiled previously (see Fig. 3).

A web server with a friendly graphical user interface was constructed to properly share the constructed MultiRM model among the research community. It takes the RNA sequence as input and returns the predicted RNA modification sites together with the key sequence contents that drive the positive predictions (Fig. 4). The statistical significance of the prediction was also provided by comparing it to the results generated from the negative sites. For online prediction, a notification email can be optionally sent to the provided email address when the job is finished. For off-line prediction, the trained MultiRM model together with Python codes can be downloaded for use on a local computer.

## Discussion

In this work, we developed a multi-label model that can simultaneously predict the twelve widely occurring RNA modifications and present the key sequence components that contributed most to the predictions.

In order to fully exploit the inherent structure of the input sequence, we experimented with three different embedding techniques on our model and found Word2vec drastically enhance its predictive capability. We also found that inputting longer RNA sequences may not necessarily lead to higher prediction accuracy. To deal with the imbalanced label problem, we implemented OHEM and Uncertain Weighting strategies. It was encouraging to find the overall performance of our MultiRM model achieved is better than the classic machine learning model XGBoost and some start-of-the-art multi-label learners.

We carefully curated the training and test data of our predictive models using only high-quality epi-transcriptome profiles generated from multiple orthogonal technologies and multiple studies. Given the discrepancy among the epi-transcriptome profiling results of multiple technologies, this remedy is of crucial importance to ensure the robustness and reliability of the predictor.

To best share the newly constructed RNA modification site predictor, a web server was constructed. Besides a friendly user interface and detailed documentation for online usage, it also hosts the trained MultiRM models and the Python codes, which may be downloaded to local computers for command-line usage.

Although MultiRM is capable of predicting twelve different types of RNA modifications, it is currently restricted to humans only and has not been extended to other model organisms. This is mainly due to the lack of availability of base-resolution epi-transcriptome profiling data for other organisms. It would be intriguing to test the performance of MultiRM on other species, such as mice and yeast, as well as to incorporate new emerging transcriptome modifications, e.g., ac4C^48,49^ and hm^5^C^50^ when such data is available in the future.

It is important to note that, MultiRM currently does not consider the distinct abundance of different RNA modifications. So even under the same setting (*p*-value cut-off), the proportion of false-positive predictions varies substantially between the more abundant RNA modifications (such as m^6^A) and the less abundant ones (such as m^1^A), i.e., with a much higher false-positive rate for the less abundant RNA modifications. The problem is partially due to the limited consistency among existing biotechnologies for profiling RNA modifications. For example, while more than 10,581 m^5^C sites were reported from bisulfite sequencing, only 617 and 1084 m^5^C sites were reported by AzaIP and miCLIP, respectively^32^, probably due to their different technical preference and sensitivity. We provided in Supplementary Fig. 5 the performance metrics of the proposed MultiRM model on unbalanced sample size, which reflects our current knowledge of the modifications’ distribution in real-world; however, it is important to note that the number of RNA modification sites collected are strongly affected by the detection sensitivity of biotechnology and the available experimental data rather than their true abundance. More reliable false discovery rate control would be desired in the future when the overall abundance of these RNA modifications is more readily available.

Our model revealed for the first time the positive associations among all the twelve RNA modifications in terms of their sequence preference. It should be of immediate interest to study the key regulatory regions of general RNA modifications and epi-transcriptome regulation. Of equal interest is their dynamic cross-talk under different biological conditions, which calls for the integrated prediction of condition-specific epi-transcriptome profiles when such data is more abundantly available. For example, by extending related studies^25–27^ under the multi-label learning framework. Previously, due to the lack of epi-transcriptome datasets in matched biological conditions, cross-talk of multiple RNA modifications was mainly studied via the expression level of relevant RNA modification enzymes^51^. Although the interactions among different RNA modifications can partially be revealed from enzyme-based analysis, it is important to note that the known enzyme genes have multiple biological functions other than writing or erasing RNA modifications, which may contaminate the results. In contrast, direct analysis of the epi-transcriptome profiles is likely to be more reliable. With the advances in deep learning approaches, it should be possible to dig more deeply and unveil the cooperative RNA modification interactions and their soft sequence syntax, as has been done in cooperative transcription factor regulation^52^.

## Methods

### Raw data and preprocessing

The development of an RNA modification site prediction model typically requires transcriptome-wide profiling data at base-resolution for training and testing purposes. A selection of datasets was made, prioritizing those derived from multiple studies and generated with different technologies. Data generated from flawed technologies (such as ordinary RNA bisulfite sequencing) or methods (such as ordinary MeRIP-seq combined with motif search) were not used.

We ultimately obtained 20 epi-transcriptome profiles generated from 15 different base-resolution technologies for 12 different types of RNA modifications (m^6^A, m^1^A, m^5^C, m^5^U, m^6^Am, m^7^G, Ψ, I, Am, Cm, Gm, and Um), as shown in Table 5. To the best of our knowledge, our data covered all the widespread RNA modifications that can be profiled transcriptome-wide at base resolution. Special attention was paid to construct the most reliable negative control data (non-modified nucleotides) for the predictor. Negative sites were randomly selected from the unmodified bases of the same transcript containing the positive sites.

**Table 5:** List of base-resolution epi-transcriptome profiling data.

Full name	Short name	Original base	Site# (%)	Technology (or Database)	GEO Accession
N6-methyladenosine	m^6^A	A	65,178(40.08%)	m^6^A-CLIP-seq	GSE71154
					GSE86336
				miCLIP	GSE98623
					GSE63753
Pseudouridine	ψ	U	3137(1.93%)	ψ-seq	GSE60047
				Pseudo-seq	GSE58200
				CeU-Seq	GSE63655
				RBS-Seq	GSE90963
1-Methyladenosine	m^1^A	A	16,380(10.07%)	miCLIP	GSE97908
				m^1^A-MAP	GSE102040
				RBS-Seq	GSE90963
				m^1^A-seq	GSE97419
					GSE70485
N6,2′-O-dimethyladenosine	m^6^Am	A	2447(1.5%)	miCLIP	GSE122948
					GSE78040
					GSE63753
2′-O-methyladenosine	Am	A	1591(0.98%)	Nm-seq	GSE90164
				RMBase^54^	–
2′-O-methylcytidine	Cm	C	1878(1.15%)	Nm-seq	GSE90164
				RMBase^54^	–
2′-O-methylguanosine	Gm	G	1471(0.90%)	Nm-seq	GSE90164
				RMBase^54^	–
2′-O-methyluridine	Um	U	2253(1.39%)	Nm-seq	GSE90164
				RMBase^54^	–
5-Methylcytidine	m^5^C	C	12,936(7.95%)	BS-seq	GSE122260
7-Methylguanosine	m^7^G	G	1036(0.64%)	m^7^G-seq	GSE112276
5-Methyluridine	m^5^U	U	1696(1.04%)	miCLIP & FICC-seq	GSE109183
Inosine	I	A	52,618(32.36%)	RADAR^53^	–

Am, Cm, Gm, and Um are sometimes combined together as 2′-O-methylation (or Nm).

The Inosine (I) sites were collected from the RADAR database^53^, while Am, Cm, Gm, and Um sites were collected from RMBase^54^ supplemented by those reported by Nm-seq^55^. When there are motifs representing modifications, i.e., the DRACH motif of m^6^A and the BCA motif of m^6^Am, the motif was used to further restrict the positive and negative data of the corresponding modification. For m^6^A, because the reliability of the existing large number of base-resolution studies using various techniques needs to be examined, a total of 87,616 m^6^A sites identified previously (Supplementary Table 4) were only used indirectly by excluding them from the negative m^6^A sites.

In the end, over 300k sites were collected. We separated the RNA sequences in each class (corresponding to a type of RNA modification) into three sets, i.e., the training set, validation set, and test set. Here, the training set is unbalanced across different classes (modification types), i.e., the number of sites is different for different RNA modifications, while the validation and test set have balanced samples with sizes 150 and 50, respectively. In general, the hyper-parameters were optimized based on the validation sets, while the reported final prediction performance was achieved on the test set. Traditionally, *K*-fold cross-validation is used to mitigate overfitting in many Machine Learning problems, especially for those who have small training data. This is because using *K*-fold to validate a model can better estimate how the results of the model will be generalized to an independent data set, especially in a limited dataset, where a small test cannot reflect the entire distribution of the data. In our scenario, however, we have ~300k training data in total. Consequently, a 5% testing/validation set will already give us a good estimate.

### Embeddings

To develop high-precision computational methods, it is essential to wisely represent or embed sequence data. Suppose we have raw data ${R}_{0}={\{{x}^{m}\}}_{m=1}^{M}$ where $M$ is the number of sequences and each ${x}^{m}\in {{\mathbb{R}}}^{L}$ is an RNA sequence. Each entry ${x}_{i}^{m},i=1,2,\ldots ,L$ at position $i$ is taking value from the alphabet $\sum =\{A,C,G,U,N\}$ from a sequence of constant length $L$. We considered the following three schemes to map the RNA sequences ${R}_{0}$ into the embedding spaces $R^{\prime}$ .

Traditionally, one-hot^56,57^ is a simple yet very effective encoding method to represent sequence data. For each RNA sequence ${x}_{i}^{m}\in \sum$, we map it by $f:\sum \mapsto {{\mathbb{R}}}^{4}$, where $f(A)=(1,0,0,0)$, $f(C)=(0,1,0,0)$, $f(G)=(0,0,1,0)$, $f(U)=(0,0,0,1)$ and $f(N)=(0,0,0,0)$. After that, ${R}_{0}$ goes to ${R}_{{\rm{onehot}}}={\{{x}^{m}\}}_{m=1}^{M}$ where each ${x}^{m}\in {{\mathbb{R}}}^{4L}$ is an RNA sequence.

Because of the ability to capture long-range interaction, Hidden Markov Model is suitable for modeling sequence data. It has been successfully applied by Seq2vec^58^, which uses a neural network to speed up the parameterization in HMM. It built a nonlinear feature embedding $f:\sum \mapsto {{\mathbb{R}}}^{n}$ which transforms each RNA sequence ${x}^{m}$ into an n-dimensional vector. Besides, $f$ is a composition of two nonlinear operators $b:{\sum }^{L}\mapsto {{\mathbb{R}}}^{d\ast L}$ and $g:{{\mathbb{R}}}^{d\ast L}\mapsto {{\mathbb{R}}}^{n}$, such that, $f({x}^{m})=g(b({x}^{m}))\,$and $b({x}^{m})=[{\mu }_{1},{\mu }_{2},\ldots ,{\mu }_{L}]$, where each ${\mu }_{l}$ summaries the potential long-range interaction of different positions in ${x}^{m}$, and $g$ will aggregate interaction information and a fixed dimensional embedding for the entire dataset. In our work, we added the HMM layer before the recurrent module and multi-label module, forming an end-to-end solution from training to predicting modification sites.

Since first invented by Mikolov in 2013, Word2vec^59^ has enhanced the performance of various NLP tasks. As a statistical language model, it follows skip-gram and continuous-bag-of-words (CBOW) architectures and uses neural networks to learn word embeddings based on context relationships. We trained our own RNA embeddings by treating each RNA sequence as a sentence and the k consecutive RNA nucleotides (k-mer) as words in that sentences. Mathematically, we define a mapping from single nucleotides to the vector representation of k-mers as $f:{\sum }^{L}\mapsto {Y}^{L-k+1}$, which are then fed into the neural networks to obtain n-dimensional embedding. It has been demonstrated in Gene2vec^15^ that 3-mers has the best predictive performance on m^6^A sites. Therefore, in our work, we follow that 3-mers convention to embed our input data. More specifically, a 3-nt sliding window moves over 1001-nt sample sequences with stride 1-nt to create sequences of 999 words with overlap. Each word corresponds to an index from the collection of all possible 3-mer combinations (104 different combinations in our training data). Then, Word2vec was implemented by Gensim package^60^ with a five-word-long window of neighboring words to learn the inherent relationship and generate a 300-dimensional feature vector. Finally, each embedded RNA sequence is converted into a 999 by 300 matrix.

### Model design

In this work, two types of DNN architecture, convolutional neural networks (CNNs) and recurrent neural networks (RNNs) were utilized to learn the sequence features of RNA modifications. Specifically, long short-term memory (LSTM) was implemented to account for possible long-range dependencies of the features.

The model mainly consists of three parts (see Fig. 1). The first module is an embedding module that takes the one-hot encoding of RNA sequences as input and embeds them by three different embedding techniques. Then, each embedding is fed into an LSTM and a Bahdanau Attention Layer^39^. Both the hidden states ${c}_{i}$ inside of the LSTM layer and the learned feature representation ${y}_{i}$ are aggregated to obtain attention weights for each target class. Then 12 different context vectors are obtained by calculating the inner product of ${y}_{i}$ and each attention weight. It is expected that these vectors can well compress the important information needed for each prediction branch. The Multi-label module, which contains 12 parallel FC layers with the ReLU activation function, maps each context vector to the probability of each modification simultaneously. Dropout layers are used to mitigate overfitting. The whole model is optimized by weighting binary cross-entropy loss in different tasks.

Importantly, to assess the contribution of the embedding methods used in our model, we exploited three variations of embedding. For one-hot encoding, a CNN has added ahead and executed as part of the embedding module. Since the original one-hot encoding of RNA sequences is a sparse input, CNNs will help extract sequence patterns in a dense manner and generate high dimensional representations of these motifs. Besides, pooling layers are utilized to trim less informative features.

It is worth noting that the data among different labels (classes) was highly imbalanced, i.e., the number of sites varied substantially between the more abundant modifications (such as m^6^A and I) and the less abundant ones (such as Am and Gm). Traditionally, this problem has been addressed in two ways. One way is to alter the original imbalanced data to balance it using an oversampling algorithm like SMOTE^61^. Another potentially more effective way is to weigh the loss of each class at the end of the network. Since our inputs are RNA sequences that were rigorously generated, generating artificial RNA sequences may degrade the credibility of the original dataset. Therefore, we focused on the second option. As a benchmark, we first used a constant weight for each task based on the Effective Numbers of Samples^62^. Subsequently, we designed our multi-label model to be self-paced by learning the weights of each task during training and only back-propagated the samples with higher loss (OHEM)^35^ so that it could jointly prioritize tasks and samples through the whole training process. We also tested the performance of focal loss^63^, which basically down-weights the loss assigned to well-classified samples on the common binary cross-entropy loss.

### Evaluation metrics

After training on the training set, we evaluated our model on the validation set and test set. The classification performance was characterized by the receiver-operating characteristic (ROC) and assessed by the area under the ROC curves (denoted as AUROC or AUC), which is a non-parametric indicator that reflects the performance of a model.

Specifically, we calculated two types of AUC: AUC_b_ and AUC_m_. AUC_b_ was computed from the positive and the corresponding negative samples of each modification (with b representing binary), while AUC_m_ was calculated using all other labels, including all other modifications and all the unmodifiable nucleotides as the negative (with m representing multiple). Consequently, AUC_b_ represents the goodness of prediction for one particular modification versus its original non-modifiable base, e.g., m^1^A vs. A, while AUC_m_ generalizes to all other cases, including other modifications and non-modifiable bases. Although AUC_m_ is the matched evaluation scheme for multi-label learning tasks, AUC_b_ was provided to maintain comparability with existing works, most of which perform binary classification with the unmodifiable original nucleotide as the negative samples. It is worth noting that nucleotides other than the original one were also considered when calculating AUC_m_. Because it is straightforward to make correct negative predictions according to the non-original nucleotide of a particular modification (for example, the nucleotide C cannot form m^6^A), AUC_m_ is substantially higher than AUC_b_.

The widely adopted assessment metrics, including sensitivity (Sn), specificity (Sp), accuracy (Acc), and Matthews correlation coefficient (Mcc), were also implemented to assess the prediction performance, and can be expressed as,

1 $${\rm{Sn}}=\frac{{\rm{TP}}}{{\rm{TP}}+{\rm{FN}}},\,{\rm{Sp}}=\frac{{\rm{TN}}}{{\rm{TN}}+{\rm{FP}}}$$

2 $${\rm{Acc}}=\frac{{\rm{TP}}+{\rm{TN}}}{{\rm{TP}}+{\rm{FP}}+{\rm{FN}}+{\rm{TN}}}\,{\text{and}}$$

3 $${\rm{Mcc}}=\frac{{\rm{TP}}\times {\rm{TN}}-{\rm{FP}}\times {\rm{FN}}}{\sqrt{({\rm{TP}}+{\rm{FP}})({\rm{TP}}+{\rm{FN}})({\rm{TN}}+{\rm{FP}})({\rm{TN}}+{\rm{FN}})}}$$

where TP represents true positive samples, TN represents true negative samples, FP represents false-positive samples, and FN represents false negative samples. The optimal threshold was chosen based on maximized G-mean^40^ for each class to classify the positive and negative samples of a particular modification. These metrics were all calculated under the binary classification scenario, i.e., using the positive and negative samples of a specific modification, and are thus comparable to the reported performance in most of the existing works on RNA modification site prediction. AUC_b_ is used as the primary evaluation metric for its nonparametric characteristics and comparability to the reported performance of related works in the existing literature.

### Statistical significance

The statistical significance of a predicted probability is assessed by an upper bound of the p-value, indicating how extreme the observed probability is among all the occurrences of the same nucleotide. It is calculated from the relative ranking of the putative RNA modification sites, i.e., if only 1% of nucleotides report a probability larger than a specific site, then the upper bound of the *p*-value of this site is 0.01. This is used as the cut-off of the prediction. However, it is important to note that the cut-off controls only type I errors. Even with the same *p*-value cut-off, the proportion of false-positive predictions are still substantially different between the more abundant RNA modifications and the less abundant ones.

### Interpretation

In addition to the accurate prediction of RNA modifications, it is often appealing to grasp the idea behind the model’s prediction. In our model, we used attention weights and IG to explain visually how the model makes specific decisions. Specifically, we focused on what our model valued most while making different predictions and

acquired the nucleotide which contributed most while making the positive prediction through attention weights and IGs.

Bahdanau attention^40^ was originally introduced as a solution to handle the long input sequences of the sequence-to-sequence model. Here, we transplanted it to our method by mapping the input RNA sequences to 12 context vectors. Since it has access to the entire input RNA sequences and is capable of picking out specific elements from the sequence to produce output, the mechanism thus gives the model freedom to focus and place more or less attention on the relevant nucleotide of the input RNA sequence for each prediction task as needed. Consequently, by visualizing the attention weights, which represent the weights of each nucleotide of the input RNA sequences in each prediction task, we can identify the most critical part of the input sequences in our model while making different predictions.

By calculating the gradient of an output neuron with respect to its input, the gradient-based attribution method can reflect how much the input features contributed to a particular output through the networks. In our work, we used an attribution method called IGs^39^. Here, the target neuron of interest is the classification layer of each modification. The IG computes the averaged gradients of the output neuron when the input varies along a linear path from a baseline or reference to the input. It measures the contribution of each input to modification prediction and assigns higher scores to important nucleotides in the input sequences. Based on the contribution scores in each input nucleotide position, we visualized the attribution map as sequence logos where the height represents the importance of that position in the prediction. The size of nucleotides in a positive direction represents an important level in predicting the appearance of RNA modifications.

Visualization of the attribution maps of each input sequence for a specific RNA modification not only gives the important positions while making positive predictions but also reveals the potential target motif (or recurring patterns) of its corresponding modifications. In order to calculate the consensus motif contributed most for each RNA modification, following a previous study^64^, we accumulated the attribution values in each position corresponding to all true positive samples with prediction scores in the top 10%. Then, for each sample, we search for the top k motifs across the attribution map by taking the highest mean scores in sliding windows of the desired length, removing its neighborhood, and repeating again for the next motif. After multiple sequence alignment, UMAP^65^ was used to embed the top-ranking motifs and DBSCAN^66^ was used to cluster these embedded motifs. Finally, we aggregated these motifs in each cluster by calculating their PWM and visualized them using sequence logos.

### Baseline performance

Since tree-based classification algorithms often have the best off-the-shelf accuracy for many bioinformatics problems^29,56^, in this work, we compared our model with two gradient-boost decision trees, i.e., XGBoost^37^ and CatBoost^38^. XGBoost has been widely used for bioinformatics predictions. CatBoost is used here because it has built-in techniques to deal with categorical variables thus avoided the extra step to convert nucleotides to one-hot encoding in this problem. The gradient-boost decision trees were used as multiclass classifiers over all 13 classes, including the 12 wide occurring RNA modifications and the non-modifiable nucleotide class. To generate optimal results, we searched the hyper-parameters of each method by coordinated decent, and the optimized choices were provided in Supplementary Table 2.

### Attention-based DNNs

We trained our attention-based DNNs (see Model Design subsection) over the short RNA sequences of 101-bp, 51-bp, or 21-bp windows. For Word2vec (see Embeddings section), we pre-trained the RNA 3-mers over 1001-bp sequence and then extracted the short RNA subsequence of the corresponding length from it. For CNN and HMM, we designed them in an end-to-end manner with the one-hot encoding of RNA sequences as the input. During training, we used a mini-batch size of 128 as the input and trained on 1 NVIDIA RTX 2080Ti over 100 epochs. In addition, we used an Adam optimizer^67^ and a mini-batch size of 128 during training. Meanwhile, exponential and cosine annealing^68^ learning rate decay were implemented for suitable models, and early stopping^69^ was introduced when the generalization loss increased in five successive epochs to prevent overfitting on the training data. Finally, the validation set was used to search for the best hyper-parameters for a single model, and the test set was used to choose the best model among various models with their best performance.

### Reporting summary

Further information on research design is available in the Nature Research Reporting Summary linked to this article.

## Supplementary information

Supplementary Information

Reporting Summary

## Data Availability

All data used in this study were already publicly available in the GEO database, RMBase, and RADAR database. In GEO database, m^6^A data can be collected from GSE71154, GSE86336, GSE98623 and GSE63753; Pseudouridine (Ψ): GSE60047, GSE58200, GSE63655 and GSE90963; m^1^A: GSE97908, GSE102040, GSE90963, GSE97419 and GSE70485; m^6^Am: GSE122948, GSE78040 and GSE63753; 2′-O-methyladenosine (Am, Cm, Gm, Um): GSE90164; m^5^C: GSE122260; m^7^G: GSE112276; m^5^U: GSE109183. 2′-O-methyladenosine data was also collected from the RMBase database under 2′-O-Me[http://rna.sysu.edu.cn/rmbase/2-O-Methylation.php] tag. Inosine data was collected from the RADAR database. All accession codes for data used are found in Table 5. All processed sequence data is freely available on the MultiRM web server at www.xjtlu.edu.cn/biologicalsciences/multirm. Detailed data profile information can be found in Supplementary Materials. All data are available from the authors upon reasonable request.

## Code availability

The deep learning framework was implemented using Pytorch, and the Python codes can be freely accessed at https://github.com/Tsedao/MultiRM^70^. The user-friendly MultiRM web server developed for easy access to our approach via a graphical user interface is at www.xjtlu.edu.cn/biologicalsciences/multirm.
