# Supplementary material for: Attention-based multi-label neural networks for integrated prediction and interpretation of twelve widely occurring RNA modifications
Source: Nat Commun. 2021 Jun 29;12:4011. doi: 10.1038/s41467-021-24313-3 (PMC8242015; doi:10.1038/s41467-021-24313-3)
Supplement: Supplementary file 1 — Supplementary Information [file 41467_2021_24313_MOESM1_ESM.pdf]

Attention-based multi-label neural networks for integrated prediction and interpretation of twelve widely occurring RNA modifications

Supplementary Table 1 AUROC scores of XGBoost with different lengths of input sequences

**Length (bp)**	**Am**	**Cm**	**Gm**	**Tm**	**m^{1}A**	**m^{5}C**	**m^{5}U**	**m^{6}A**	**m^{6}Am**	**m^{7}G**	**Ψ**	**I**	**Mean**
25	0.6296	0.8516	0.9456	0.7340	0.8224	0.8896	0.9184	0.7868	0.7992	0.6744	0.8264	0.6072	0.7904
51	0.6536	0.8124	0.9500	0.7608	0.8604	0.9096	0.9300	0.8120	0.8668	0.6796	0.7956	0.6112	0.8035
101	0.6136	0.8196	0.9332	0.7460	0.8648	0.8936	0.9352	0.8016	0.8688	0.6560	0.8280	0.6292	0.7991

Note: The input length 51bp returns the best overall prediction performance.

Supplementary Table 2 Optimized hyper-parameters for each model

Model	XGBoost	CatBoost	CNN+LSTM+Attention	HMM+LSTM+Attention	MultiRM
Learning rate	0.2	0.01	0.0001-0.001	0.0001-0.001	0.0001-0.001
Learning rate decay	NA	NA	Cosine Annealing (T_max = 5) Stepwise decay learning rate by 10^{-1} every 20 epochs	Cosine Annealing (T_max = 5) Stepwise decay learning rate by 10^{-1} every 20 epochs	Cosine Annealing (T_max = 5) Stepwise decay learning rate by 10^{-1} every 20 epochs
Iterations/Epochs	1000	1000	100	20	100
Other Parameters	n_estimators:1000, max_depth:4, min_child_weight:1, max_delta_step:10, subsample:0.8, colsample_bytree:0.8, scale_pos_weight:1	max_depth:10, l2_leaf_reg:1, scale_pos_weight:100	NA	NA	NA

Supplementary Table 3 Multi-label performance on MultiRM

Metrics	Formula*	MultiRM	HMM+LSTM +Attention	CNN+LSTM +Attention	XGBoost
Precision	$\frac{1}{n}\cdot\sum_{i}^{n}\frac{\lvert T_i\cap S_i\rvert}{\lvert S_i\rvert}$	0.2199	0.2072	0.2088	0.2151
Recall	$\frac{1}{n}\cdot\sum_{i}^{n}\frac{\lvert T_i\cap S_i\rvert}{\lvert T_i\rvert}$	0.4758	0.4717	0.4717	0.4783
F1-score	$\frac{1}{n}\cdot\sum_{i}^{n}\frac{2\lvert T_i\cap S_i\rvert}{\lvert T_i\rvert+\lvert S_i\rvert}$	0.2878	0.2769	0.2796	0.2861
Hamming loss	$\frac{1}{n}\cdot\sum_{i}^{n}\frac{XOR(T_i,S_i)}{k}$	0.1288	0.1366	0.1593	0.1385

Note: *The performance metrics of multiple-label prediction performance were taken from Sorower[1], where T_i represents ground true labels of sample i, S_i represents predicted labels of sample i, and k stands for number of labels.

Supplementary Table 4 Additional known m^6A sites (87,616) excluded from the negative training data

ID	Technique	Cell line	GEO	GSM	Treatment	Ref.
1	miCLIP	HepG2	GSE73405	GSM2011453<br>GSM2011454	Heat shock	2
2				GSM2011455<br>GSM2011456	Ctrl	
3		HEK293T		GSM2011452	Heat shock	
4		HEK293T	GSE122948	GSM3489018	Ctrl	3
5				GSM3489019<br>GSM3489020	PCIF1 KO	
6		HepG2	GSE121942	GSM3450336<br>GSM3450337<br>GSM3450338<br>GSM3450339<br>GSM3450340<br>GSM3450341	SETD2 KD	4
7				GSM3450333<br>GSM3450334<br>GSM3450335	Ctrl	
8		HCT116	GSE128699	GSM3682895<br>GSM3682896	Ctrl	5
9				GSM3682897<br>GSM3682898	METTL5 KO	
10				GSM3682899<br>GSM3682900	ZCCHC4 KO	
11	MAZTER-seq	HEK293T	GSE122961	GSM3723222<br>GSM3723223<br>GSM3723224	Ctrl	6
12				GSM3723219<br>GSM3723220<br>GSM3723221	AlkBH5 OE	
13				GSM3723225<br>GSM3723226<br>GSM3723227	FTO OE	
14		ESC		GSM3723216<br>GSM3723217<br>GSM3723218	Ctrl	
15		ESC		GSM3723213<br>GSM3723214<br>GSM3723215	FTO KO	
16	m6A-REF-seq	liver	GSE125240	GSM3566983	Ctrl	7
17		brain		GSM3566979		
18		kidney		GSM3566981		

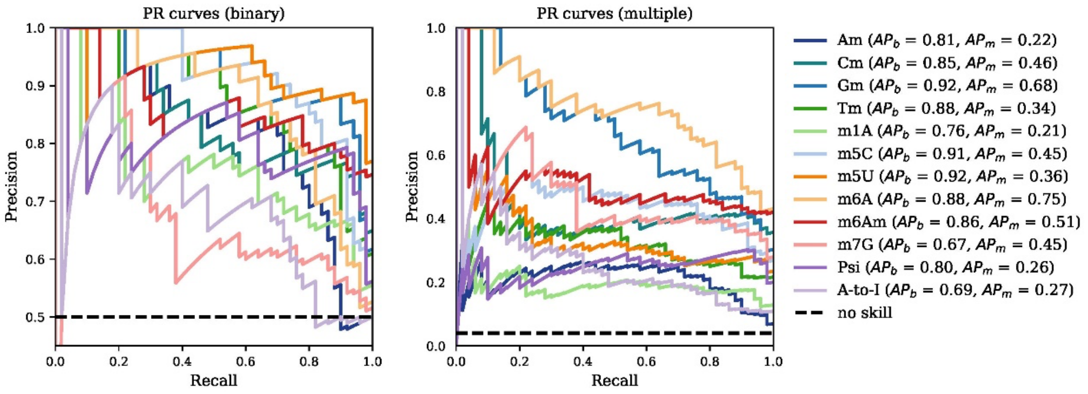

Supplementary Fig. 1 Precision-Recall Curves. Precisions and Recalls (PRs) were computed on a balanced test data. For the left figure (binary mode), PRs of each class was tested against its own negative data, forming a binary performance metric. The right one (multiple mode) selected all other classes' positive samples as its negative data while testing the performance of one class.

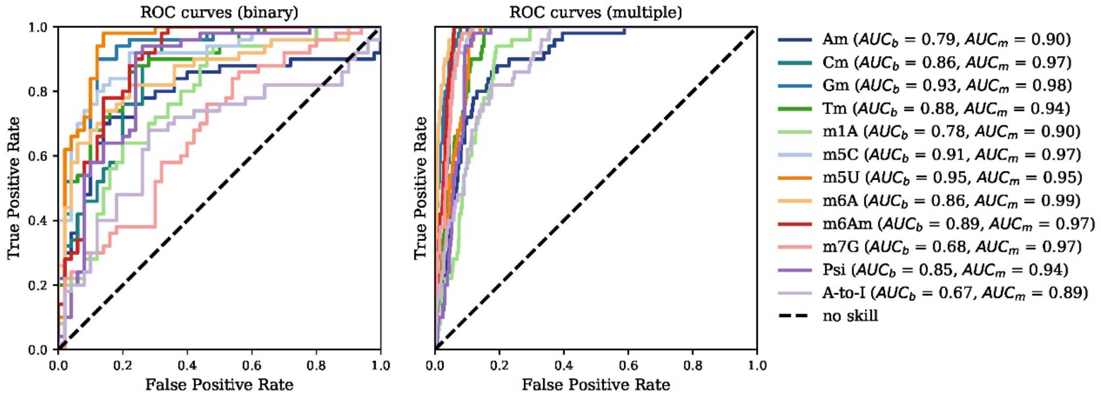

Supplementary Fig. 2 Receiver Operating Characteristic Curves. Receiver Operating Characteristic curves (ROCs) were computed on a balanced test data. For the left figure, true positive rates (TPRs) and false positive rate (FPRs) of each class was tested against its own negative data, forming a binary performance metric. The right one selected other classes' positive samples as its negative data while testing the result of one class.

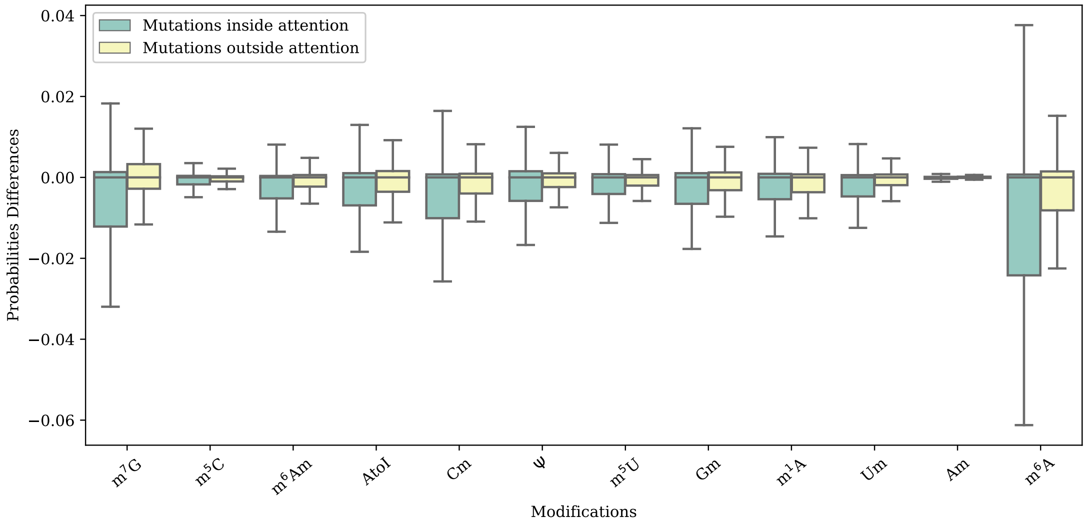

Supplementary Fig. 3 Impact of mutations on nucleotides inside and outside of attention. We calculated the difference in the probability of RNA modifications between the wild type and the randomly mutated sequences (n=9984 for each construct). In this figure, box denotes interquartile (IQR) ranges, centers mark medians and whiskers extend to 1.5 IQR from the quartiles. Result suggests that the mutations inside of the attention regions have greater impact on RNA modifications compared with those outside of attention. Mutations happen within the attention are more likely to lead to the gain or loss of the corresponding RNA modification site.

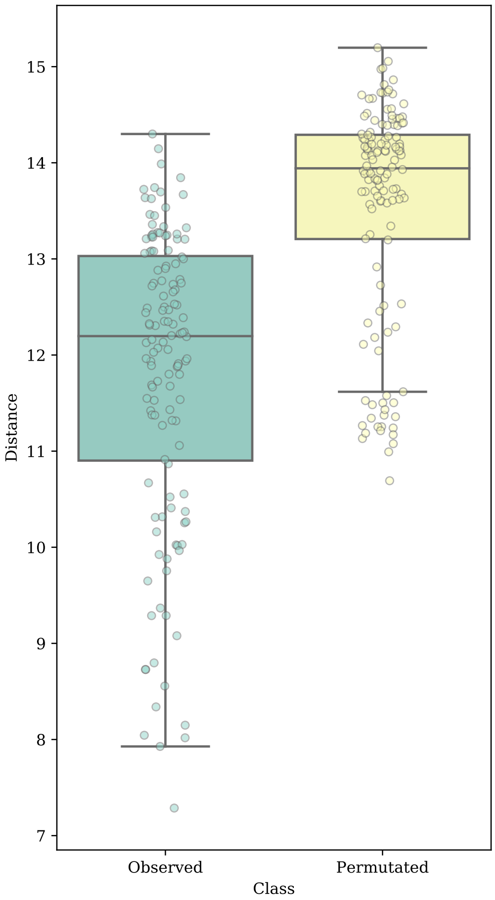

Supplementary Fig. 4 Distance between two arbitrary RNA modifications. Data are represented as boxplots, where box denotes interquartile (IQR) ranges, centers mark medians and whiskers extend to 1.5 IQR from the quartiles. When testing the aggregation effects between two arbitrary RNA modifications, we considered only the genes that carry both two types of RNA modification and refer to them as the testing modification and the reference modification. We firstly calculated the median distances from testing modification sites to their closest reference modification sites (n=132), and then random permutation them on mRNAs and recalculated these distances as random control (n=132). As shown from the above figure, there exist clear aggregation effects among different RNA modifications. This observation suggests again that there exist certain regions on RNA that are likely to be regulated by various RNA modifications. It may be important to mention that we could not rule out of the possibility of experimental bias.

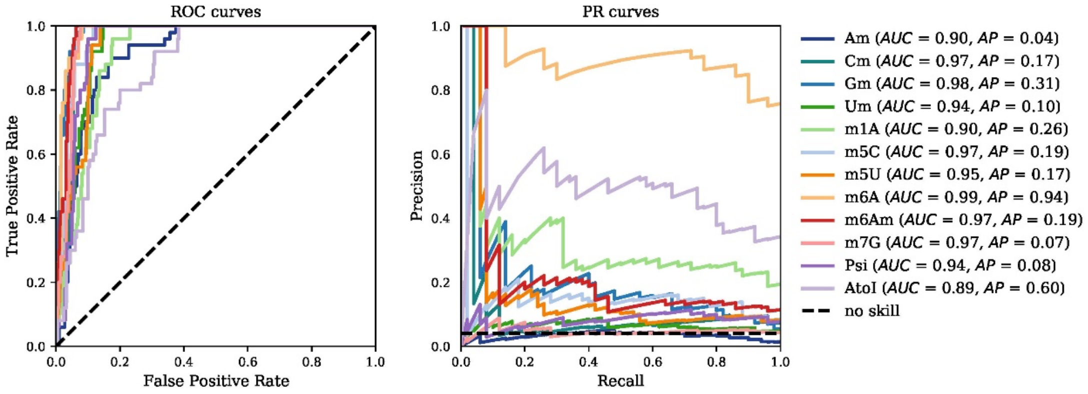

Supplementary Fig. 5 Performance on unbalanced datasets. Precisions and Recalls (PRs) and Receiver Operating Characteristic curves (ROCs) were computed on an unbalanced test data. The ratio of the positive and negative test data in each class follows original collected data, which is assumed to reflect modifications' distribution in real-world.

Reference

1. Sorower, M. S. A literature survey on algorithms for multi-label learning. *Oregon State University, Corvallis* **18**, 1-25 (2010).
2. Meyer, K. D.*, et al.* 5' UTR m^{6}A Promotes Cap-Independent Translation. *Cell* **163**, 999-1010 (2015).
3. Boulias, K.*, et al.* Identification of the m^{6}Am Methyltransferase PCIF1 Reveals the Location and Functions of m^{6}Am in the Transcriptome. Mol Cell 75, 631-643 e638 (2019).
4. Huang, H., et al. Histone H3 trimethylation at lysine 36 guides m^{6}A RNA modification co-transcriptionally. Nature 567, 414-419 (2019).
5. van Tran, N., et al. The human 18S rRNA m6A methyltransferase METTL5 is stabilized by TRMT112. Nucleic acids research 47, 7719-7733 (2019).
6. Garcia-Campos, M. A., et al. Deciphering the "m^{6}A Code" via Antibody-Independent Quantitative Profiling. Cell 178, 731-747 e716 (2019).
7. Zhang, Z., et al. Single-base mapping of m^{6}A by an antibody-independent method. Science advances 5, eaax0250 (2019).
