# Supplementary material for: Attention-based multi-label neural networks for integrated prediction and interpretation of twelve widely occurring RNA modifications
Source: Nat Commun. 2021 Jun 29;12:4011. doi: 10.1038/s41467-021-24313-3 (PMC8242015; doi:10.1038/s41467-021-24313-3)
Supplement: Supplementary file 2 — Reporting Summary [file 41467_2021_24313_MOESM2_ESM.pdf]

nature research

Corresponding author(s): Jia Meng

Last updated by author(s): May 31, 2021

Reporting Summary

Nature Research wishes to improve the reproducibility of the work that we publish. This form provides structure for consistency and transparency in reporting. For further information on Nature Research policies, see our Editorial Policies and the Editorial Policy Checklist.

Statistics

For all statistical analyses, confirm that the following items are present in the figure legend, table legend, main text, or Methods section.

n/a	Confirmed	
☐	☒	The exact sample size (*n*) for each experimental group/condition, given as a discrete number and unit of measurement
☐	☒	A statement on whether measurements were taken from distinct samples or whether the same sample was measured repeatedly
☐	☒	The statistical test(s) used AND whether they are one- or two-sided<br>*Only common tests should be described solely by name; describe more complex techniques in the Methods section.*
☒	☐	A description of all covariates tested
☐	☒	A description of any assumptions or corrections, such as tests of normality and adjustment for multiple comparisons
☐	☒	A full description of the statistical parameters including central tendency (e.g. means) or other basic estimates (e.g. regression coefficient) AND variation (e.g. standard deviation) or associated estimates of uncertainty (e.g. confidence intervals)
☐	☒	For null hypothesis testing, the test statistic (e.g. *F*, *t*, *r*) with confidence intervals, effect sizes, degrees of freedom and *P* value noted<br>*Give P values as exact values whenever suitable.*
☒	☐	For Bayesian analysis, information on the choice of priors and Markov chain Monte Carlo settings
☒	☐	For hierarchical and complex designs, identification of the appropriate level for tests and full reporting of outcomes
☐	☒	Estimates of effect sizes (e.g. Cohen's *d*, Pearson's *r*), indicating how they were calculated

Our web collection on statistics for biologists contains articles on many of the points above.

Software and code

Policy information about availability of computer code

Data collection	Data was collected from public databases: GEO database, RMBase and RADAR. Therefore, no code was utilized to collect data.
Data analysis	All scripts and code used in this work have been made available in Github: https://github.com/Tsedao/MultiRM (http://doi.org/10.5281/zenodo.4851898).<br>Softwares used in this work: CatBoost 0.23.2, XGBoost 0.90, UMAP 0.4.6, DBSCAN in Scikit-Learn 0.22.1.<br>Webserver used in this work: DREME [https://meme-suite.org/meme/doc/dreme.html] STREME [https://meme-suite.org/meme/tools/streme] TOMTOM [https://meme-suite.org/meme/tools/tomtom]<br>MultiRM web server: www.xjtlu.edu.cn/biologicalsciences/multirm

For manuscripts utilizing custom algorithms or software that are central to the research but not yet described in published literature, software must be made available to editors and reviewers. We strongly encourage code deposition in a community repository (e.g. GitHub). See the Nature Research guidelines for submitting code & software for further information.

Data

Policy information about availability of data

All manuscripts must include a data availability statement. This statement should provide the following information, where applicable:

- Accession codes, unique identifiers, or web links for publicly available datasets
- A list of figures that have associated raw data
- A description of any restrictions on data availability

All data used in this study were already publicly available in the GEO database, RMBase and RADAR database. In GEO database, m6A data can be collected from GSE71154, GSE86336, GSE98623 and GSE63753; Pseudouridine: GSE60047, GSE58200, GSE63655 and GSE9096 3; m1A: GSE97908, GSE102040, GSE90963, GSE97419 and GSE70485; m6Am: GSE122948, GSE78040 and GSE63753; 2’-O-methyladenosine (Am, Cm, Gm, Um): GSE90164; m5C: GSE122260; m7G: GSE112276;

m5U: GSE109183. All data from GEO database can be found using link in the format [https://www.ncbi.nlm.nih.gov/geo/query/acc.cgi?acc=GSE]. For example, GSE71154 can be visited through [https://www.ncbi.nlm.nih.gov/geo/query/acc.cgi?acc=GSE71154]. 2'-O-methyladenosine data was also collected from RMBase database under 2'-O-Me tag. Inosine data was collected from RADAR database. All processed sequence data is freely available on the MultiRM web server at www.xjtlu.edu.cn/biologicalsciences/multirm. Detailed data profile information can be found in Supplementary Materials. All data are available from the authors upon reasonable request. Accession codes for the data used to generate Figure 2 in manuscript and Figure 1, 2, 3, 4, 5 in Supplementary Materials are found in Table 5. The weights used to generate Figure 3 in manuscript can be found in the GitHub's repository [https://github.com/Tsedao/MultiRM/tree/master/Weights/MultiRM].

Field-specific reporting

Please select the one below that is the best fit for your research. If you are not sure, read the appropriate sections before making your selection.

☒ Life sciences ☐ Behavioural & social sciences ☐ Ecological, evolutionary & environmental sciences

For a reference copy of the document with all sections, see nature.com/documents/nr-reporting-summary-flat.pdf

Life sciences study design

All studies must disclose on these points even when the disclosure is negative.

Sample size

The sample size of the data used in this study is the amount of available base-resolution epitranscriptome profiling data for all 12 RNA modifications we were able to collect until the beginning of the work, except for N6-Methyladenosine (m6A). For m6A, only the data used in the published predictors [1, 2, 3, 4] is considered in this study.

1. Zhou, Y., Zeng, P., Li, YH., Zhang, Z. & Cui, Q. SRAMP: prediction of mammalian N6-methyladenosine (m6A) sites based on sequence-derived features. Nucleic Acids Res 44, e91 (2016).
2. Chen, K., et al. WHISTLE: a high-accuracy map of the human N6-methyladenosine (m6A) epitranscriptome predicted using a machine learning approach. Nucleic Acids Res 47, e41 (2019).
3. Zou, Q., Xing, P., Wei, L. & Liu, B. Gene2vec: gene subsequence embedding for prediction of mammalian N6-methyladenosine sites from mRNA. RNA (New York, NY) 25, 205-218 (2019).
4. Chen, Z., et al. Comprehensive review and assessment of computational methods for predicting RNA post-transcriptional modification sites from RNA sequences. Brief Bioinform 21, 1676-1696 (2020).

Data exclusions

A total of 87616 reported m6A sites were excluded from the data used. Details can be found in Supplementary Table 4. The reason behind this data exclusion is that only the data used in previous studies [1, 2, 3, 4] is used to maintain comparability with existing works. In addition, duplicate sites identified through various technologies were excluded.

1. Zhou, Y., Zeng, P., Li, YH., Zhang, Z. & Cui, Q. SRAMP: prediction of mammalian N6-methyladenosine (m6A) sites based on sequence-derived features. Nucleic Acids Res 44, e91 (2016).
2. Chen, K., et al. WHISTLE: a high-accuracy map of the human N6-methyladenosine (m6A) epitranscriptome predicted using a machine learning approach. Nucleic Acids Res 47, e41 (2019).
3. Zou, Q., Xing, P., Wei, L. & Liu, B. Gene2vec: gene subsequence embedding for prediction of mammalian N6-methyladenosine sites from mRNA. RNA (New York, NY) 25, 205-218 (2019).
4. Chen, Z., et al. Comprehensive review and assessment of computational methods for predicting RNA post-transcriptional modification sites from RNA sequences. Brief Bioinform 21, 1676-1696 (2020).

Replication

The experiments were replicated at least 2 times for each setting. All attempts at replication were successful. The saved models and weights as well as hyperparameters used are available at https://github.com/Tsedao/MultiRM

Randomization

For all experiments, all sequences were randomly split into training, validation and test sets.

Blinding

The test sets used for performance measures were completely independent of data sets used for model training and validation.

Reporting for specific materials, systems and methods

We require information from authors about some types of materials, experimental systems and methods used in many studies. Here, indicate whether each material, system or method listed is relevant to your study. If you are not sure if a list item applies to your research, read the appropriate section before selecting a response.

Materials & experimental systems

n/a	Involved in the study
☒	☐ Antibodies
☒	☐ Eukaryotic cell lines
☒	☐ Palaeontology and archaeology
☒	☐ Animals and other organisms
☒	☐ Human research participants
☒	☐ Clinical data
☒	☐ Dual use research of concern

Methods

n/a	Involved in the study
☒	☐ ChIP-seq
☒	☐ Flow cytometry
☒	☐ MRI-based neuroimaging
